# Flow Cytometric-Based Analysis of Defects in Lymphocyte Differentiation and Function Due to Inborn Errors of Immunity

**DOI:** 10.3389/fimmu.2019.02108

**Published:** 2019-09-04

**Authors:** Cindy S. Ma, Stuart G. Tangye

**Affiliations:** ^1^Immunology Division, Garvan Institute of Medical Research, Sydney, NSW, Australia; ^2^Faculty of Medicine, St. Vincent's Clinical School, UNSW Sydney, Sydney, NSW, Australia; ^3^Clincial Immunogenomics Research Consortium Australia, Darlinghurst, NSW, Australia

**Keywords:** flow cytometry, B cells, T cells, immunodeficencies, phenotpye

## Abstract

The advent of flow cytometry has revolutionized the way we approach our research and answer specific scientific questions. The flow cytometer has also become a mainstream diagnostic tool in most hospital and pathology laboratories around the world. In particular the application of flow cytometry has been instrumental to the diagnosis of primary immunodeficiencies (PIDs) that result from monogenic mutations in key genes of the hematopoietic, and occasionally non-hematopoietic, systems. The far-reaching applicability of flow cytometry is in part due to the remarkable sensitivity, down to the single-cell level, of flow-based assays and the extremely user-friendly platforms that enable comprehensive analysis, data interpretation, and importantly, robust and rapid methods for diagnosing PIDs. A prime example is the absence of peripheral blood B cells in patients with agammaglobulinemia due to mutations in *BTK* or related genes in the BCR signaling pathway. Similarly, the development of intracellular staining protocols to detect expression of SAP, XIAP, or DOCK8 expedites the rapid diagnosis of the X-linked lymphoproliferative diseases or an autosomal recessive form of hyper-IgE syndrome (HIES), respectively. It has also become evident that distinct cohorts of PID patients exhibit unique “lymphocyte phenotypic signatures” that are often diagnostic even prior to identifying the genetic lesion. Flow cytometry-based sorting provides a technique for separating specific subsets of immune cells such that they can be studied in isolation. Thus, flow-based assays can be utilized to measure immune cell function in patients with PIDs, such as degranulation by cytotoxic cells, cytokine expression by many immune cells (i.e., CD4^+^ and CD8^+^ T cells, macrophages etc.), B-cell differentiation, and phagocyte respiratory burst *in vitro*. These assays can also be performed using unfractionated PBMCs, provided the caveat that the composition of lymphocytes between healthy donors and the PID patients under investigation is recognized. These functional deficits can assist not only in the clinical diagnosis of PIDs, but also reveal mechanisms of disease pathogenesis. As we move into the next generation of multiparameter flow cytometers, here we review some of our experiences in the use of flow cytometry in the study, diagnosis, and unraveling the pathophysiology of PIDs.

## Introduction

### The Role of Technological Developments in Understanding Immunology and Immunodeficiency

The application of new technologies to basic research and clinical investigations has greatly improved biochemical and molecular analyses of cellular physiology and identified defects in these processes that underpin human disease. Thus, technological advances have enabled not only fundamental discoveries of basic immunology, but also a greater understanding of disease pathogenesis, rapid diagnoses of these conditions as well as providing opportunities for the development and/or implementation of improved therapies. Great examples of this can be found in the fields of clinical immunology and immunodeficiency.

Using electrophoretic analysis of serum from a young boy with severe and recurrent bacterial infections led to the discovery by Col Ogden Bruton of the first case of agammaglobulinemia (1). Importantly, treating this patient with monthly subcutaneous infusions of concentrated human immune serum globulin prevented further infections (1). These observations established that agammaglobulinemia caused this patient's recurrent infections, and that the gammaglobulin fraction of serum contained antibodies capable of prophylactically preventing infection. Critically, this finding in a single patient—which pre-dated the discovery of B cells by more than a decade (2)—led to the identification 40 years later that mutations in *BTK*, encoding Bruton's tyrosine kinase, cause X-linked agammaglobulinemia (XLA) (3).

Extending this work by Bruton, the advent of serological reagents capable of reacting with specific lymphocyte subsets—such as polyclonal antisera raised against surface Ig molecules—enabled the identification of B cells in the peripheral blood of healthy individuals, and the absence of Ig-expressing cells in XLA (4–7). Importantly, XLA patients were found to have near-normal frequencies of precursor B cells in their bone marrow (BM), the site of B-cell development (8), establishing that the very early—but not later—stages of B-cell development were intact in XLA patients. Similarly, advances in techniques to fractionate human peripheral blood leukocyte subsets by density gradient centrifugation allowed the isolation of populations enriched for B cells, T cells and monocytes. This elegant approach also revealed a stark paucity of B cells in XLA (9). These analytical approaches demonstrating an absence of peripheral blood Ig-expressing B cells in XLA patients provided a clear explanation for their agammaglobulinemia.

These serological studies using anti-Ig not only defined B cells as the cellular deficiency in XLA, but also gave greater clarity to other immune deficient conditions. For instance, investigation of X-linked or autosomal recessive (AR) severe combined immunodeficiency (SCID) revealed that, despite persistent lymphopenia and hypogammaglobulinemia, the majority (>90%) of lymphocytes in these individuals were actually B cells (7, 10). This established that these conditions were likely due to a deficiency of T cells, thus defining T^−^B^+^ SCID. Likewise, studies of males who were hypo- or agammaglobulinemic but had normal frequencies of B cells, along with T cells, delineated an X-linked PID distinct from XLA that probably represented X-linked hyper-IgM syndrome (6, 7).

### Monoclonal Abs Enabled Further Delineation, and Prognosis, of Immunodeficiencies

The ability to generate immortalized cells lines (hybridomas) producing monoclonal Abs (mAbs) with defined and distinct specificities (11) led to quantum leaps in basic and clinical immunology. Thus, it quickly became possible to identify immune cell populations and subsets not only according to the differential expression of surface Ig, but also by the presence, or absence of other cell surface molecules. By using mAbs against surface markers that are coordinately expressed during B-cell development, it was found that the very few B cells present in the circulation of XLA patients resembled immature B cells in BM and were distinct from mature B cells in the peripheral blood of healthy donors (12). This finding was critical in identifying the stage at which B cell development is blocked by inactivating mutations in *BTK* (3).

This approach of studying immune cell subsets by immunofluorescent microscopy was also critical in understanding pathophysiology of HIV infection and subsequent progression to AIDS. Here, a reduction in the number of peripheral blood CD4^+^ T cells, and a corresponding inversion of the CD4:CD8 T cell ratio, became a defining clinical characteristic of individuals infected with HIV (13–16). Furthermore, the steady decline in numbers of CD4^+^ T cells in HIV infection became predictive of progression to full blown AIDS, revealing the need to longitudinally track CD4^+^ T cells as a biomarker of disease progression following HIV infection (16, 17).

### Flow Cytometry Revolutionized Immunology and the Study of Immunodeficiencies

While methodologies such as density gradient centrifugation and immunofluorescent microscopy advanced our understanding of basic immunology and disease, they were laborious and often lacked the level of sensitivity and quantitation required to make definitive interpretations of the data. By simultaneously enabling the rapid analysis of large numbers of immune cells, flow cytometry has had a profound impact on immunology (18), including its application to the study of primary and acquired immunodeficiencies.

It became possible to quickly assess the status of CD4^+^ T cells in HIV infection (17), accurately define the stages and phenotypes of B cell development in human BM and how mutations in genes such as *BTK, BLNK, IGHM, IGLL1, CD79A, CD79B, PIK3R1*, and *TCF3* differentially affect this process (3, 19, 20), and delineate distinct types of SCID due to different gene mutations according to the presence and absence of specific lymphocyte populations, such as B^+^T^−^NK^–^ SCID (*IL2RG, JAK3*), B^−^T^−^NK^+^ SCID (*RAG1, RAG2*), B^+^T^−^NK^+^ SCID (*IL7RA*), or B^−^T^−^NK^−^ SCID (*ADA*) (20, 21).

The discoveries of surface molecules that are induced on activated lymphocytes, or distinguish discrete subsets of T and B cells, also led to major advances in the discovery, diagnosis, management, and classification of PIDs (20). Thus, males with X-linked hyper IgM syndrome could be identified by the inability of their CD4^+^ T cells to upregulate expression of functional CD40L following anti-CD3/CD28 or PMA/ionomycin-mediated activation (22, 23). Common variable immunodeficiency (CVID) due to *ICOS* mutations was discovered by the identification of a small number of patients whose T cells lacked ICOS expression following *in vitro* stimulation (24). The finding that CD27 is expressed on human memory, but not naïve, B cells (25, 26) enabled an entirely new stratification system of CVID that could reliably classify patients with various pathologies (27, 28).

**Figure 1:**
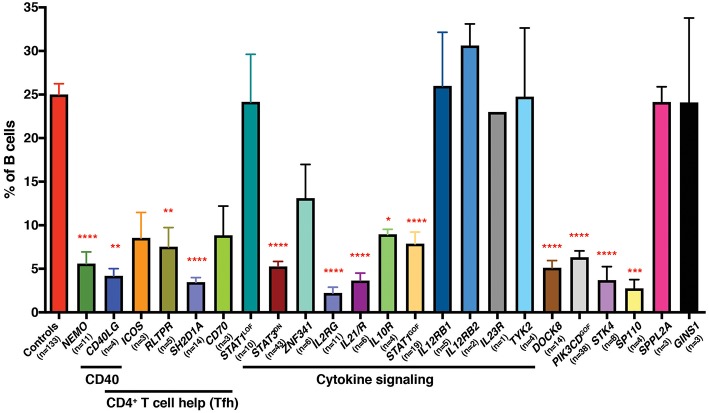
Impact of inborn errors of immunity on the generation of memory B cells. PBMCs from the indicated numbers of healthy donor controls or from individuals with pathogenic mutations in the indicated genes were stained with mAbs against CD19, CD20, CD10, and CD27. The proportions of B cells exhibiting a CD19^+^CD10^−^CD27^+^ memory phenotype was determined by flow cytometric gating and analysis. Significant differences were determined by ANOVA (^*^p < 0.05; ^**^p < 0.01; ^***^p < 0.001; ^****^p < 0.0001). The age range of the healthy donors is from birth (cord blood) to ~65 years old. These data are compiled from findings previously reported in the following publications: (29–37, 39–44).

We have also exploited this finding, together with the availability of patients with PIDs, to identify non-redundant molecular and cellular requirements for the generation and/or maintenance of memory B cells in humans. Thus, mutations that disrupt (i) CD4^+^ T cell/B cell interactions and thus delivery of CD4^+^ T cell-mediated B cell help (e.g., loss of function [LOF] *CD40L, ICOS, CD40, NEMO, SH2D1A, RLTPR* [*CARMIL2], CD27*/*CD70*), (ii) cytokine signaling particularly through IL-21 and STAT3 (i.e., *IL21, IL21R, IL2RG, ZNF341, IL10R* LOF; *STAT3* dominant negative [DN]; *STAT1* gain of function [GOF]), or (iii) other intracellular signaling pathways (*STK4, DOCK8, SP110* LOF; *PIK3CD* GOF) all reduce memory B cells (defined as CD19^+^CD20^+^CD10^−^CD27^+^ cells) in affected individuals (29–41) (Figure 1). Similar studies performed by other investigators have established that signaling via CARD11/BCL10/MALT1 (45), CD19/CD81 (46), and NIK/NFKB2 (47, 48) are also key regulators of the generation and/or maintenance of human memory B cells. Importantly, this approach also established that, for instance, IL12Rβ1/2, IL-23R, TYK2, and STAT1 signaling (32, 42), nor SPPL2A (43) or GINS1 (44), are required for generating and/or maintaining the memory B cell pool in humans (Figure 1).

**Figure 2:**
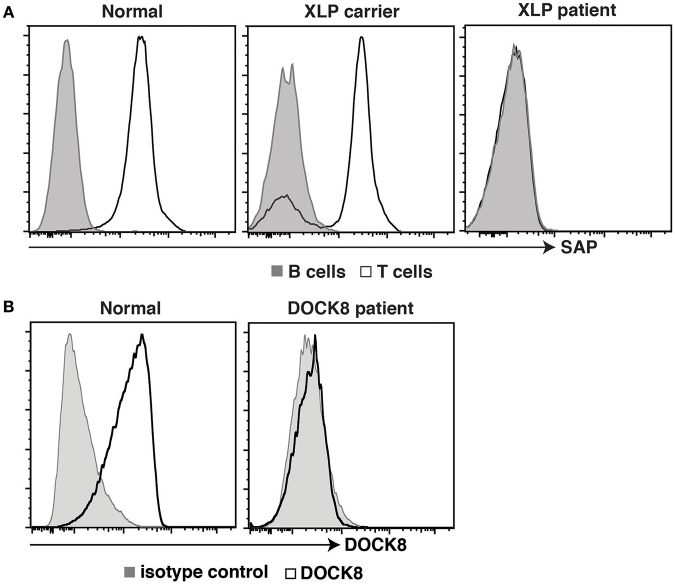
Intracellular staining for SAP and DOCK8 in controls, patients, and female carriers. **(A)** PBMCs from healthy donors, female carriers of the XLP trait, or individuals with pathogenic mutations in *SH2D1A*, or were stained with mAbs against CD3, CD20, and SAP. Expression of SAP in B cells (tinted histogram) and T cells (open histogram) was then determined. **(B)** PBMCs from healthy donors, or from an individual suspected of having DOCK8-deficiency were stained with an isotype control Ab (tinted histogram) or a mAb against DOCK8 (open histogram). Expression of DOCK8 in lymphocytes was then determined. These data are compiled from findings previously reported in the following publications: (37, 57, 58).

Further advances in flow cytometric methodologies enabled detection of intracellular proteins (49), as well as post-translational modifications to proteins involved in cell signaling (50). These developments were also embraced by the clinical immunology field to facilitate rapid diagnosis of PIDs and discover patients with novel immune defects. The ability to quantify expression of SAP, XIAP, BTK, FOXP3, and DOCK8 proteins by intracellular staining and flow cytometric analysis expedited detection and diagnosis of patients with X-linked lymphoproliferative disease (XLP) type 1 (LOF mutations in *SH2D1A*), XLP-2 (LOF mutations in *XIAP/BIRC4*), XLA (BTK), IPEX (*FOXP3*), and an AR form of HIES (*DOCK8*), respectively (51–56) (Figure 2). Furthermore, this facilitated the identification of female carriers of some X-linked traits, such as XLP-1, XLP-2, and XLA (51, 55–57, 59) (Figure 2), as well as the discovery of somatic reversion in XLP-1 (60), DOCK8-deficiency (54), and leukocyte adhesion deficiency-1 due to mutations in *ITGB2* encoding CD18 (61, 62). Similarly, the detection of intracellular phosphorylated STAT proteins in response to cytokine-mediated stimulation of lymphocyte populations has been developed as a functional screen to identify individuals with LOF mutations in *IL10R, IL12RB1*, or *IFNGR1* (63–65), or GOF mutations in *STAT1* (66). Importantly, this technique has been applied to discover patients with novel inborn errors of immunity, including LOF mutations in *IL21R* (67), *IL6ST* (68), and *IL6R* (69). Clinical flow cytometry has also played a valuable role in studying PIDs affecting innate immunity. Assessing respiratory burst in phagocytes using oxidation of fluorescent probes such as dihydrorhodamine 123 (DHR) following leukocyte activation *in vitro* is the gold standard for diagnosing individuals with either X-linked or AR forms of chronic granulomatous disease (70), as well as females carriers of the X-linked form of this condition (59). Lastly, the use of cell permeable fluorescent dyes such as carboxyfluorescein succinimidyl ester (CFSE), Cell trace violet and Cell trace yellow, to label intracellular molecules and then track the dilution and concomitant reduction in fluorescence intensity of these dyes with each cell division (71, 72) has enabled detailed analysis of the role of cell division in lymphocyte differentiation and how these events can be uncoupled or compromised in various PIDs (29–31, 33, 35–37, 40, 58, 60, 73–76).

However, there are important caveats to consider when using flow cytometry to determine potential molecular causes of PIDs. While the vast majority of gene variants found to cause PIDs abolish protein expression, and thus a lack of the encoded protein can be used as a surrogate to confirm the pathogenicity of a mutation, several variants do not affect protein expression and are pathogenic due to them being LOF. An example of this can be found in one of the original descriptions of mutations in *CD40LG*, which reported detectable expression of CD40L on activated CD4^+^ T cells from one patient with X-linked hyper-IgM syndrome despite the presence of a predicted inactivating mutation (23). Similarly, pathogenic mutations have been identified in *SH2D1A* (77), *DOCK8* (78), *BTK* (55, 56), and *IL10RA* (65) in patients with XLP, AR HIES, XLA and very early onset inflammatory bowel disease, respectively, yet expression of the encoded proteins is unaffected. Despite this, flow cytometry was still valuable in some of these cases to establish the LOF nature of variants that did not affect protein expression. Here, quantifying binding of soluble CD40 to the surface of activated CD4^+^ T cells established that a specific mutation in *CD40LG* that preserved protein expression, as determined flow cytometrically using an anti-CD40L mAb, was unable to bind CD40L (23). Likewise, LOF of expressed but mutant IL-10RA was confirmed by demonstrating an inability of IL-10 to induce phosphorylation of STAT3 in PBMCs from this patient (65). Thus, like all aspects of research and clinical immunology, awareness of the limitations of specific assays needs to be borne in mind, and multiple approaches adopted to ensure the most accurate and clinically-actionable results are obtained. But the contribution of flow cytometry to clinical medicine remains central and unquestioned.

### Delineation of Human Peripheral Blood Lymphocytes by Flow Cytometry

To study the impact of gene mutations on the human immune system of individuals with PIDs, it is important to first be able to identify distinct populations of immune cells in peripheral blood (PB), as this is the predominant source of cells that is readily obtainable from patients. In line with this, mAbs directed against a myriad of cell surface markers can be used to identify, isolate, and characterize distinct *in vivo*-generated immune cell subsets (Table 1). Of the main conventional lymphocyte populations CD3, CD4, CD8, CD20, and CD56 have been used to identify total CD3^+^ T, CD4^+^ helper T (Th), CD8^+^ cytotoxic T, CD20^+^ B, and CD3^−^CD56^+^ natural killer (NK) cells (Table 1). In addition to this, unconventional T cell subsets, which contribute to both innate and adaptive immune responses, have been identified within the T cell compartment as CD3^+^Vα24^+^Vβ11^+^ NKT, CD3^+^γδ^+^αβ^−^ γδ T and CD3^+^Vα7.2^+^CD161^+^ mucosal associated invariant T (MAIT) cells (79) (Table 1).

**Table 1:** Immune cell populations identified in human peripheral blood according to specific phenotypes.

**Immune cell**	**Surface markers**
CD4^+^ T CELLS	
Total CD4^+^ T cells	CD3^+^CD4^+^
Naïve	CD3^+^CD4^+^CCR7^+^CD45RA^+^
Central memory	CD3^+^CD4^+^CCR7^+^CD45RA^−^
Effector memory	CD3^+^CD4^+^CCR7^−^CD45RA^−^
Treg	CD3^+^CD4^+^CD25^hi^CD127^lo^FoxP3^+^
Tfh	CD3^+^CD4^+^CD45RA^−^CXCR5^+^
Th1	CD3^+^CD4^+^CD45RA^−^CXCR5^−^CXCR3^+^
Other Th cells including Th2 and Th9 cells	CD3^+^CD4^+^CD45RA^−^CXCR5^−^CXCR3^−^CCR6^−^
Th17	CD3^+^CD4^+^CD45RA^−^CXCR5^−^CCR6^+^
CD8^+^ T CELLS	
Total CD8^+^ T cells	CD3^+^CD8^+^
Naïve	CD3^+^CD8^+^CCR7^+^CD45RA^+^
Central memory	CD3^+^CD8^+^CCR7^+^CD45RA^−^
Effector memory	CD3^+^CD8^+^CCR7^−^CD45RA^−^
TEMRA	CD3^+^CD8^+^CCR7^−^CD45RA^+^
B CELLS	
Total CD20^+^ B cells	CD20^+^
Transitional	CD20^+^CD10^+^CD27^−^
Early transitional	CD20^+^CD10^+^CD27^−^CD21^lo^CD44^−/lo^
Late transitional	CD20^+^CD10^+^CD27^−^CD21^+^CD44^+^
Naïve	CD20^+^CD10^−^CD27^−^IgM^+^IgD^hi^
Memory	CD20^+^CD10^−^CD27^+^
IgM memory	CD20^+^CD10^−^CD27^+^IgM^hi^IgD^±^
IgG memory	CD20^+^CD10^−^CD27^+^IgG^+^
IgA memory	CD20^+^CD10^−^CD27^+^IgA^+^
Plasmablasts/cells	CD20^±^CD19^+^CD38^hi^CD27^hi^
Atypical/aged memory B cells	CD19^hi^CD21^lo^Tbet^+^CD11c^+^FCRL5^+^
Innate-like lymphocytes	
NK cells	CD3^−^CD56^+^
NKT cells	CD3^+^Vα24^+^Vβ11^+^
MAIT cells	CD3^+^Vα7.2^+^CD161^+^
γδ T cells	CD3^+^Vαβ^−^Vγδ^+^

Importantly, differential expression of specific cell surface markers can be used to determine the maturation status of distinct subsets of CD4^+^ and CD8^+^ T and B cells. Specifically CCR7 and CD45RA delineate naive (CD45RA^+^CCR7^+^), central memory (T_CM_; CD45RA^−^CCR7^+^), and effector memory (T_EM_; CD45RA^−^CCR7^−^) CD4^+^ T cells, and naive (CD45RA^+^CCR7^+^), T_CM_ (CD45RA^−^CCR7^+^), T_EM_ (CD45RA^−^ CCR7^−^), and revertant memory (T_EMRA_; CD45RA^+^CCR7^−^) CD8^+^ T cell populations (80, 81) (Table 1). The CD4^+^ T cell compartment can also be resolved into different “helper” subsets based on expression of CD25, CD127, CD45RA, CXCR5, CXCR3, and CCR6. As such, CD4^+^ T cell populations corresponding to regulatory T cells (Tregs; CD25^hi^CD127^lo^), T follicular helper (Tfh; CD45RA^−^CXCR5^+^), Th1 (CD45RA^−^CXCR5^−^CXCR3^+^CCR6^−^), Th2 (CD45RA^−^CXCR5^−^CXCR3^−^CCR6^−^), and Th17 (CD45RA^−^CXCR5^−^CXCR3^−^CCR6^+^) subsets can be identified in PB of healthy individuals (32, 81, 82).

CD20^+^ human B cells can be divided into transitional (CD10^+^CD27^−^), naïve (CD10^−^CD27^−^), and memory (CD10^−^CD27^+^) subsets by their differential expression of CD10 and CD27 (25, 26, 83, 84). Early/immature and late/mature subsets of transitional B cells can also be identified according to differential expression of CD21 or CD44 (35, 83, 84). Furthermore, memory B cells can remain IgM^hi^ or undergo Ig isotype switching to become IgG- or IgA-expressing cells (25, 26) (Table 1). Plasmablasts (CD19^+^CD38^hi^CD27^hi^CD20^lo^) can also be detected, though these cells persist at very low frequencies in the PB at the basal state (85). A population of CD19^hi^CD21^lo^ B cells can also be detected within the B cell compartment. These CD19^hi^CD21^lo^ have been referred to as “atypical” and/or “aged memory” B cells, and have been associated with both health and disease (86–88) (Table 1). Thus, on one hand they have been proposed as being plasmablast precursors that are rapidly re-activated and differentiate into plasmablasts during anamnestic immune responses to specific Ag (86). On the other hand they have been considered to be pathogenic in the setting of chronic infection (e.g., HIV, malaria, Hepatitis B) as they are “exhausted” and unable to clear these pathogens, or self-reactive in Ab-mediated autoimmune disease (SLE, RA, Sjogren's syndrome) (27, 87, 88).

### Insights Into Disease Pathogenesis in PIDs

Over the past two decades, our flow cytometric-based studies of various PIDs have provided substantial insight and understanding into the non-redundant roles of specific genes, molecules, and signaling pathways in the development, differentiation and effector function of human B cells, CD4^+^ T cells, CD8^+^ T cells and innate-like lymphocytes. These findings have not only identified critical requirements for establishing robust primary and long-lived immunity against various pathogens, but have elucidated mechanisms underlying infectious susceptibility in the setting of these PIDs, and defined specialized functions of discrete subsets of immune cells host defense. Some of our key findings from these studies are summarized below:

#### Autosomal Dominant Hyper IgE Syndrome Due to STAT3 Mutations

AD HIES is characterized by recurrent opportunistic bacterial (*Staphylococcal*) and fungal (*Candida* sps.) infections, recurrent cyst-forming pneumonia and impaired generation of Ag-specific Abs following vaccination or infection, despite dramatically increased levels of serum IgE (89). Affected individuals also present with non-immune connective tissue defects such as broad facial features, high palate, retention of primary teeth, hyperextensibility, scoliosis, osteoporosis, and recurrent fractures (89). AD-HIES was found to result from heterozygous germline DN mutations in *STAT3* (STAT3^DN^) (90, 91). Examination of PBMCs from these patients has revealed a “lymphocyte phenotype signature” that is distinct from healthy donors.

**Figure 3:**
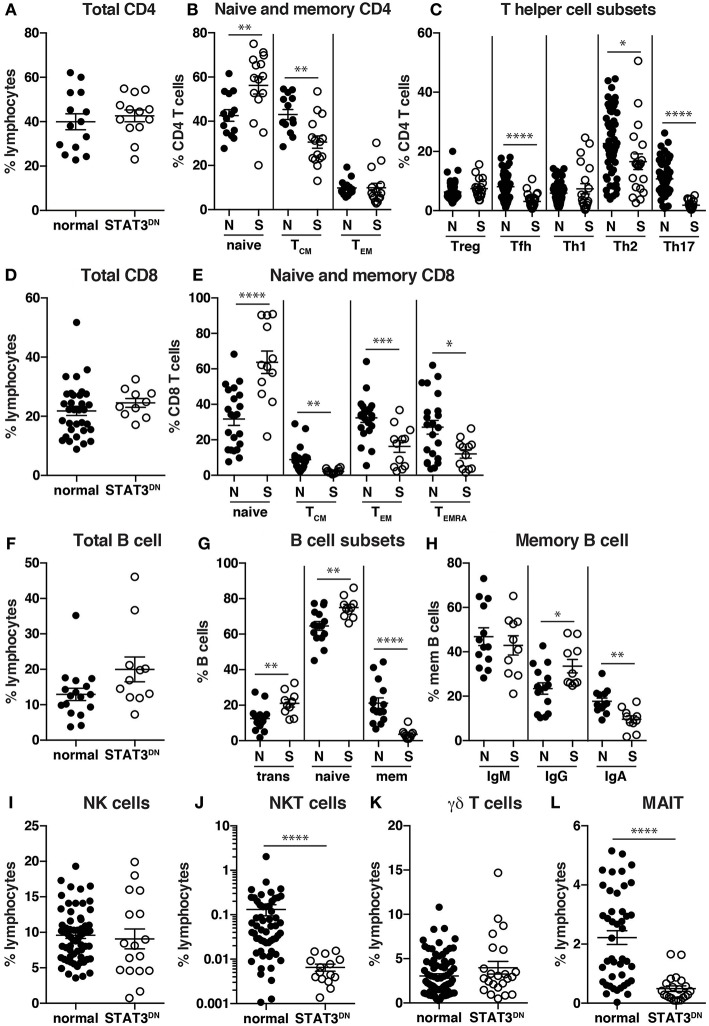
Identification of immune cell populations in STAT3^DN^ patients. **(A)** Total CD4 T cells, **(B)** naïve, central memory (T_CM_), effector memory (T_EM_) CD4^+^ T cells, **(C)** regulatory T cells (Treg) T follicular helper (Tfh) cells, Th1, Th2, Th17 CD4^+^ T cells, **(D)** total CD8^+^ T cells, **(E)** naïve, T_CM_, T_EM_, and revertant memory (T_EMRA_) CD8^+^ T cells, **(F)** total B cells, **(G)** transitional, naive and memory B cells, **(H)** IgM, IgG and IgA memory B cells, **(I)** NK cells, **(J)** NKT cells, **(K)** γδ T cells and **(L)** mucosal associated invariant T (MAIT) cells were identified in normal donors (N) and STAT3^DN^ patients (S). Each point represents a different sample and horizontal line represents the average; statistics were performed in Prism using *t*-test (^*^p < 0.05; ^**^p < 0.01; ^***^p < 0.001; ^****^p < 0.0001). These data are compiled from findings previously reported in the following publications: (31, 32, 73–75, 92, 93).

**Figure 4:**
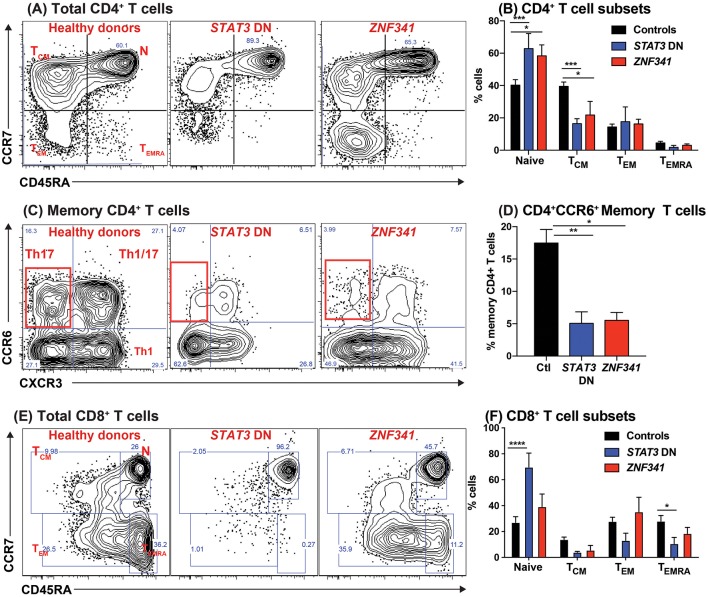
Recessive mutations in *ZNF341* phenocopy clinical and cellular defects due to autosomal dominant *STAT3* mutations to cause AR HIES. PBMCs from healthy donors or from individuals with pathogenic *STAT3* DN or *ZNF341* LOF mutations were stained with mAbs against CD4, CD8, CD45RA, CCR7, CXCR3, CCR6, CXCR5, CD127, and CD25. **(A,B)** Total CD4^+^ T cells were identified as CD4^+^CD8^−^ cells, and then proportions of naïve, T_CM_, T_EM_, and T_EMRA_ cells were determined. **(C,D)** the memory compartment (CD4^+^CD45RA^−^) of CD4^+^ T cells was analyzed to quantify the proportions of cells with a CXCR3^−^CCR6^+^ Th17-, CXCR3^+^CCR6^−^ Th1-, CXCR3^+^CCR6^+^ Th1/17-, and CXCR3^−^CCR6^−^ Th2-type phenotype. **(E,F)** Total CD8^+^ T cells were identified as CD4^−^CD8^+^ cells, and then proportions of naïve, T_CM_, T_EM_, and T_EMRA_ cells were determined. The contour plots in **(A,C,E)** are representative of 1 healthy donor, and 1 patient each with mutations in *STAT3* or *ZNF341*. The graphs represent the mean ± sem of CD4^+^ and CD8^+^ T cell subsets detected in the PB of 17 healthy donors, 8 *STAT3*^*DN*^ patients or 4 *ZNF341*-deficient patients. Significant differences were determined by ANOVA (^*^p < 0.05; ^**^p < 0.01; ^***^p < 0.001; ^****^p < 0.0001). These data are compiled from our findings previously reported in Beziat et al. (40).

Specifically, while there were normal frequencies of total CD4^+^ T cells in STAT3^DN^, we found increases in proportions of naïve and decreases in proportions of T_CM_ cells (Figures 3A,B, 4A,B) (32). Further investigations into CD4^+^ T cell subsets revealed a significant decrease in CXCR5^+^ Tfh and CCR6^+^ Th17 cells and to a lesser extent an increase in the CXCR5^−^CXCR3^−^CCR6^−^ memory population (32, 92, 94, 95), which contains Th2 cells, in patients with STAT3^DN^ mutations (Figures 3C, 4C,D). Frequencies of total CD8^+^ T cells are comparable in healthy donors and STAT3^DN^ patients (Figure 3D), however STAT3^DN^ patients have an increase in naïve and a corresponding decrease in T_CM_, T_EM_, and T_EMRA_ CD8^+^ T cells (Figures 3E, 4E,F) (73). Examination of the B cell compartment of STAT3^DN^ patients also revealed stark differences to healthy donors. Despite normal frequencies of total B cells (Figure 3F), they tended to be more immature as revealed by an increase in transitional and naïve B cells and a concurrent decrease in memory B cells (Figures 1, 3G) (31, 32, 74). Interestingly, despite the severe reduction in memory B cells, those that do develop in STAT3^DN^ patients have undergone Ig isotype switching, albeit with a trend toward IgG and away from IgA (Figure 3H) (31, 74).

In regards to innate-like lymphocytes, the frequencies of NK and γδ T cells in the PB of STAT3^DN^ patients is normal (Figures 3I,K). However, we found a severe reduction in NKT and MAIT cells in the absence of intact STAT3 signaling (Figures 3J,L) (93, 95). Taken together this analysis revealed a distinct phenotype for STAT3^DN^ lymphocytes compared to healthy controls. This lymphocyte signature has not only aided in the identification of potential STAT3^DN^ patients, but also provided valuable insights into disease susceptibility and a cellular and molecular explanation for the clinical features of STAT3 deficiency. For instance, CCR6^+^ Th17 cells are implicated in protective immunity against fungal infections (81, 95). Thus, the severe reduction in Th17 cells in STAT3^DN^ patients explains their extreme susceptibility to *Candida albicans* and subsequent chronic mucocutaneous candidiasis. Furthermore, the significant reductions in memory B cells and Tfh cells due to STAT3 deficiency are likely to account for defects in long-lived humoral immunity in STAT3^DN^ patients (95).

While these findings established critical roles for STAT3 signaling in the generation and/or maintenance of various populations of effector lymphocytes, they did not directly identify the upstream STAT3-activating cytokine(s) required for these processes. However, this has now been achieved by the identification and analysis of PID patients with inactivating mutations in specific cytokines or their receptors that signal through STAT3. Thus, IL-21/IL-21R/STAT3 signaling is required for establishing the pool of memory B cells (31, 32, 74, 95) and NKT cells (93, 95), IL-23R/IL-12Rβ1/STAT3 (but not IL-12Rβ2) signaling is necessary for MAIT cells (42, 93), and IL-23R/IL-12Rβ1/STAT3 and IL-21/IL-21R/STAT3 signaling likely co-operate to generate Th17 cells (32, 42, 95).

#### Mutations in the Novel Transcription Factor ZNF341 Underlie a Form of Autosomal Recessive HIES That Phenotypically and Functionally Resembles STAT3 Deficiency

Recently, 2 studies identified 19 patients with an AR form of HIES who essentially clinically phenocopied individuals with STAT3^DN^ mutations (40, 96). The molecular lesion underlying this form of recessive HIES was found to be bi-allelic mutations in the novel transcription factor *ZNF341*. The link between ZNF341 and STAT3 function was provided by the finding that ZNF341 binds to the *STAT3* promoter and regulates STAT3 expression. Consequently, ZNF341-deficient patients have low levels of *STAT3* mRNA and protein and poor responses following stimulation with STAT3-activating cytokines (40, 96).

**Figure 5:**
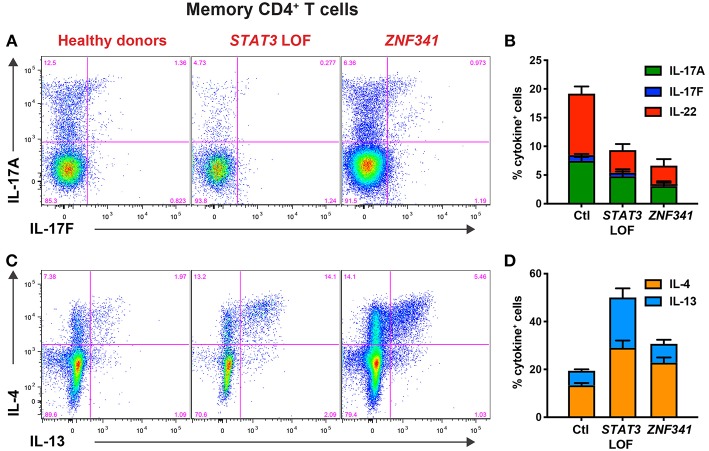
CD4^+^ T cell defects detectable by immunophenotyping of *STAT3*^DN^ and *ZNF341*-deficient patients correlate with compromised function determined flow cytometrically *in vitro*. Sort-purified CD4+ memory T cells isolated from healthy donors or individuals with pathogenic *STAT3* DN or *ZNF341* LOF mutations were cultured *in vitro* with anti-CD2/CD3/CD28 mAb conjugated to beads. Following 5 days, the cells were harvested, washed, and then restimulated with PMA/ionomycin for 6 h, with Brefeldin A being added for the final 4 h of culture. Cells were then harvested, fixed, permeabilised, and stained with fluorescently-labeled mAbs specific for **(A,B)** IL-17A and IL-17F, or **(C,D)** IL-4 and IL-13. The proportions of cytokine-expressing cells were then determined by flow cytometric analysis. Contour plots **(A,C)** are representative of 1 healthy donor, and 1 patient with mutations in *STAT3* or *ZNF341*. The graphs **(B,D)** represent the mean ± sem of CD4^+^ memory T cells expressing the indicated cytokine from 23–28 healthy donors, 6–7 *STAT3*^*DN*^ patients or 4–6 *ZNF341*-deficient patients. These data are compiled from our findings previously reported in Beziat et al. (40).

When PBMCs from ZNF341-deficient patients were examined, we identified a lymphocyte signature very similar to that of STAT3^DN^ patients. The CD4^+^ T cell compartment was comprised of increased frequencies of naïve and decreased frequencies of T_CM_ cells (Figures 4A,B) (32, 40). This could be further broken down to reveal decreases in CCR6^+^ Th17 (Figures 4C,D) and CXCR5^+^ Tfh cells and increases in Th2 cells (40, 96). The paucity of Th17 phenotype cells in ZNF341- and STAT3-deficient patients (i.e., CD4^+^CCR6^+^CXCR3^−^ memory T cells) was confirmed functionally by demonstrating by flow cytometry reductions in proportions of their memory CD4^+^ T cells that expressed intracellular IL17A, IL17F, and IL22 (Figures 5A,B) (32, 40, 92), canonical cytokines produced by Th17 cells (81, 95). Similarly, ZNF341-deficient and STAT3^DN^ patients had increased proportions of memory CD4^+^ T cells expressing the characteristic Th2 cytokines IL-4 and IL-13 (Figures 5C,D) (40), consistent with the finding of increased CD4^+^CD45RA^−^CXCR5^−^CCR6^−^CXCR3^−^ memory cells (Figure 3C), as well as the hyper-IgE phenotype and Th2-associated pathologies, in these individuals (40, 95, 96). However, in contrast to STAT3^DN^ patients, the CD8^+^ T cell compartment in ZNF341-deficient patients was relatively comparable to that of normal donors (Figures 4E,F) (40, 73). ZNF341-deficient patients have similar decreases in memory B cells as STAT3^DN^ patients (Figure 1), and their few memory B cells are predominantly IgG^+^ rather than IgA^+^ (31, 40, 74). When populations of innate-like T cells were examined, ZNF341-deficient patients had comparable frequencies of γδ T and NKT cells, but fewer MAIT cells than healthy donors (40). In contrast to STAT3, ZNF341 is likely to be important for NK cell development, as these cells are significantly decreased in ZNF341-deficient patients compared to healthy controls (40).

#### DOCK8-Deficiency

Dedicator of cytokinesis 8 (DOCK8) deficiency is a combined immunodeficiency caused by AR LOF mutations in *DOCK8* (97). This disorder is characterized by recurrent cutaneous viral, bacterial and fungal infections, increased serum IgE levels, and severe atopic disease, including food-induced anaphylaxis (97). Similar to SAP expression in XLP, the use of mAbs to detect DOCK8 expression has been crucial for the diagnosis of DOCK8-deficient patients (37, 53, 97) (Figure 2). Intracellular flow cytometry for DOCK8 expression has also detected somatic reversion in these patients (54).

DOCK8-deficient patients also have a unique lymphocyte phenotype, with decreased frequencies of CD4^+^ T cells, but normal frequencies of CD8^+^ T cells, resulting in an inverted CD4:CD8 ratio compared to healthy donors (37, 58, 76). When DOCK8-deficient CD4^+^ and CD8^+^ T cells were further investigated, we found a decrease in naïve CD4^+^ and CD8^+^ T cells and a corresponding increase in CD4^+^ T_EM_ and CD8^+^ T_EM_ and T_EMRA_ populations compared to healthy donors (37, 58, 76). Furthermore, memory T cells in DOCK8-deficiency contained reduced frequencies of CD27^+^, CD28^+^, and CD127^+^ and higher frequencies of CD57^+^, CD95^+^, and PD1^+^ cells, indicating these cells had undergone premature exhaustion or senescence compared to their counterparts in healthy donors (37, 58, 76). There were also reductions in frequencies of CD4^+^CCR6^+^ memory T cells in DOCK8-deficient patients, suggesting a role for DOCK8 in Th17 cells (37, 58, 78). This loss of CCR6^+^ Th17 cells also provides a cellular explanation for increased susceptibility of DOCK8-deficient patients to infections with *Candida* sp. (81, 95). The B cell compartment in DOCK8-deficient patients comprises normal frequencies of total and transitional B cells, however there are increases in naïve and decreases in memory B cells compared to healthy donors (37, 98), thus highlighting a requirement for DOCK8 for B cell differentiation. DOCK8-deficient patients were also found to have decreased αβ T, NKT, and MAIT cells, normal frequencies of NK cells, but increased γδ T cells (37). Importantly, this cellular phenotype has been able to predict individuals with AR HIES who may have pathogenic mutations in *DOCK8*, and provide explanations for clinical features of this condition (37, 58, 76).

#### X-Linked Lymphoproliferative Disease

XLP-1 is a rare X-linked PID whereby affected males present with extreme sensitivity to disease resulting from Epstein-Barr virus (EBV) infection. Following exposure to EBV, XLP patients develop severe infectious mononucleosis, leading to often-fatal hemophagocytic lymphohistiocytosis (99). XLP-1 patients also exhibit hypogammaglobulinemia and a heightened risk of developing B-cell lymphoma, both of which occur independently of exposure to EBV (99). XLP-1 results from LOF mutations in *SH2D1A*, encoding signaling lymphocytic activation molecule (SLAM)-associated protein (SAP) (20). SAP is a small intracellular adaptor protein that binds to tyrosine-based motifs in the intracellular domain of SLAM family members and regulates signaling downstream of these receptors (99). The availability of mAbs specific for SAP has been instrumental in expanding our knowledge of this condition. Thus, flow cytometry has (i) accurately defined the cell types that express SAP (predominantly T, NK, and NKT cells, but rarely B cells), (ii) offered a rapid and sensitive diagnostic tool to detect not only XLP patients whom lack SAP expression, but also female carriers of the XLP trait who have bimodal SAP expression in their T and NK cells (Figure 2) (57), and (3) revealed lymphocyte defects in XLP-1, thereby providing insight into disease pathogenesis. XLP patients have a paucity for CD27^+^ memory B cells (Figure 1), and the few memory B cells detected express IgM, thereby indicating an inability to undergo class switching to express IgG or IgA (29, 30). Within the CD4^+^ T cell compartment, XLP patients have a comparable frequency of naïve, T_CM_ and T_EM_ populations to healthy donors (29). Although XLP patients have a normal frequency of Tfh cells (100, 101), the inability of these cells to support B cell differentiation in an *in vitro* helper assay indicates defective B-helper function (30). Together, these observations identify defective CD4^+^ T cell help as the cellular basis for hypogammaglobulinemia in XLP-1 patients, rather than a B-cell intrinsic defect. This is indeed consistent with the fact that B cells do not express SAP (57, 60) and XLP B cells function normally when provided with the correct T-dependent stimuli *in vitro* (30).

CD8^+^ T cells from XLP patients are skewed toward T_EM_ and T_EMRA_ cells, usually at the expense of naïve T cells. Interestingly, by utilizing intracellular flow cytometry to detect SAP expression, we were able to show that some XLP patients undergo somatic reversion and a population of SAP^+^ CD45RA^−^CCR7^−^ T_EM_ cells is detectable within their CD8^+^ T cells (60). These reverted SAP^+^ CD8^+^ T cells in XLP patients could respond to EBV and kill-EBV infected B cells, suggesting they were able to provide protective immunity against ongoing EBV infection (60). These functional features were detected by concomitantly tracking proliferation and degranulation of EBV-specific CD8^+^ T cells (60), further illustrating the utility of flow cytometry in establishing functionality of immune cells in PIDs.

XLP patients also lack NKT cells, revealing an essential role for SAP in the development of this cell type and potentially implicating NKT cells in some of the clinical manifestations of XLP such as impaired anti-viral and anti-tumor immune responses (102). Consistent with this essential role of SAP in NKT cell development, female XLP carriers undergo X chromosome inactivation within NKT cells, but not T or NK cells, resulting in all NKT cells in these carriers expressing only the WT allele (57, 102).

## Final Remarks

Flow cytometry has been central to enhancing our understanding of PIDs. It has enabled diagnoses and provided mechanistic insights into disease pathogenesis in many PIDs. For example, the severe reduction in Th17 cells and memory B cells in *STAT3*^DN^, *ZNF341*-, and *DOCK8*-deficient patients explains susceptibility to recurrent opportunistic bacterial and fungal infections, and impaired long-lived protective humoral immunity, respectively. While our focus has been on lymphocyte populations, flow cytometry has been used to identify other populations such as monocytes (CD14^+^), dendritic cell (DC) subsets [plasmacytoid DCs (pDCs; lineage^−^HLA-DR^+^CD123^+^), CD1c^+^ myeloid DCs (CD1c^+^ mDCs; lineage^−^HLA-DR^+^CD11c^+^CD1c^+^), and CD141^+^ mDC (CD141^+^ mDC; lineage^−^HLA-DR^+^CD11c^+^CD141^+^)], and innate lymphoid cells [(ILCs), ILC1, ILC2, and ILC precursors (ILCPs)]. Beyond deep immunophenotypic analysis by examining expression of specific cell surface molecules, flow cytometry has also been used to diagnose PIDs by assessing expression of specific intracellular proteins (SAP, DOCK8, XIAP, BTK) as well as quantifying cytokine-induced STAT phosphorylation and Ag-receptor induced calcium flux. As we move toward the next generation of flow cytometers, which are capable of simultaneously detecting upward of 28 fluorochromes, the future is looking brighter, fluorescent even, in terms of the applicability of flow cytometry in the study and diagnosis of PIDs.

## Author Contributions

CM and ST designed, conceptualized, and wrote this review.

### Conflict of Interest Statement

The authors declare that the research was conducted in the absence of any commercial or financial relationships that could be construed as a potential conflict of interest. The handling editor declared a past co-authorship with the authors.
